# Oral supplementation of melatonin attenuates the onset of alcohol-related liver disease

**DOI:** 10.1007/s00109-025-02583-4

**Published:** 2025-08-07

**Authors:** Franziska Kromm, Anja Baumann, Victor Sánchez, Annette Brandt, Raphaela Staltner, Ina Bergheim

**Affiliations:** Department of Nutritional Sciences, Molecular Nutritional Science, University of Vienna, Josef-Holaubek-Platz 2, UZA II, Vienna, A-1090 Austria

**Keywords:** Ethanol, Intestinal barrier dysfunction, Tight junctions, AMPK, NO

## Abstract

**Abstract:**

Studies suggest that supplementing melatonin in pharmacological doses may attenuate the development of liver diseases including alcohol-related liver diseases (ALD) in model organisms. If melatonin at “physiological” doses achievable through the intake of foods and beverages affects the development of liver diseases, it has not yet been clarified; therefore, we assessed whether supplementing “dietary doses” of melatonin affects the development of ALD in mice. Female 6–8-week-old C57BL/6J mice were either pair-fed a liquid alcohol-enriched Lieber DeCarli diet or a control diet ± melatonin (50 ng/kg BW/day) for 6 weeks. Markers of liver damage and intestinal barrier function were assessed. Moreover, the effects of melatonin on intestinal barrier function were assessed in an ex vivo model. Supplementing melatonin significantly attenuated the development of ALD being related to lower interleukin-6 protein, NOx, and 4-hydroxynonenal protein adduct levels in liver tissue. Impairments of intestinal barrier function in small intestine in ethanol-fed mice were significantly attenuated in ethanol-fed mice treated with melatonin being associated with lower NOx and higher phosphorylation levels of AMPK. In summary, our results suggest that an oral supplementation of “dietary” doses of melatonin may dampen the development of ALD in mice.

**Key messages:**

- Supplementation of “dietary” doses of melatonin dampens the development of ALD.
- Melatonin attenuates alcohol-induced small intestinal barrier dysfunction.
- Protective role of melatonin is related to alterations of AMPK activity.

**Supplementary Information:**

The online version contains supplementary material available at 10.1007/s00109-025-02583-4.

## Introduction

Despite intensive efforts to raise awareness of the health consequences of regular, excessive alcohol consumption, the consumption of alcoholic beverages remains high in many countries, especially in Europe but also in the USA [1]. Due to its anatomical position and function in the metabolism, the liver is one of the organs that is particularly sensitive to the damaging effects of alcohol. While the number of patients suffering from metabolic dysfunction-associated steatotic liver disease (MASLD) by far outnumbers those suffering from alcohol-related liver diseases (ALD), the number of deaths attributed to excessive alcohol consumption still accounts to ^~^5.3% per year [2]. Abstinence is the therapy of choice [3]; however, relapse rates are high [4]. Therefore, delineating molecular mechanisms associated with the development, especially of the later stages of ALD, e.g., fibrosis and cirrhosis, are still at need to improve treatment strategies of ALD patients.

Melatonin (N-acetyl-5-methoxytryptamine) is synthesized not only in the pineal gland but also in the small intestine, liver, retina, lymphocytes, and melanocytes in skin [5]. Melatonin is a hormone critical in many physiological processes including the regulation of circadian rhythm, immune response and inflammation (for overview [6]). Depending on dietary habits, marked amounts of melatonin are also ingested through foods and beverages (Table 1; [7, 8]). Studies suggest that melatonin may be critical in maintaining gastrointestinal tract function and attenuating the development of liver damage in various settings including ALD [6, 9]. Indeed, results of several studies in rodents employing different models of ALD and a few human studies suggest that an oral supplementation of melatonin may attenuate the development and progression of ALD [9]. For example, it has been shown that a treatment with pharmacological doses of melatonin ranging from 10 to 50 mg melatonin/kg/day applied orally, i.v. and i.p., respectively, decreases signs of liver steatosis and inflammation in rodents being related to a significant reduction of ethanol-induced apoptosis and inflammation including a suppression of the activation of NFκB and TNFα-dependent signaling cascades in liver tissue [10, 11]. It also has been shown that supplementing melatonin may attenuate alcohol-related increases of bacterial endotoxin in blood and alterations of intestinal barrier function in rats [12]. However, molecular mechanisms underlying the protective effect of melatonin on ALD have not yet been fully clarified. Also, if effects alike are found when “physiological” amounts of melatonin, achievable through diet, are consumed, it has not been determined.

**Table 1:** Content of melatonin in different sources of food

	Type of food	Amount of melatonin	Concentrations per 100 mL or 100 g	Reference
Beverages	Beer (4–7% alcohol content)	84.6–169.7 pg/mL	8.46–16.97 ng/100 mL	[13]
	Wine	8.4–23 ng/mL	840–2300 ng/100 mL	[14]
	Orange juice (*Citrus sinensis* L. var. Navel late)	3.15–21.80 ng/mL	315–2180 ng/100 mL	[7]
	Coffee brew Coffea canephora Coffea arabica	0.06 µg/mL 0.078 µg/mL	6 µg/100 mL 7.8 µg/100 mL	[15]
Vegetables	Mushrooms (*Cantharellus cibarius)*	1400 ng/g DW	140 µg/100 g DW	For overview see [7]
	Tomato (*Solanum lycopersicum* L. cv. Optima)	249.98 ng/g DW	24.99 µg/100 g DW	
	White mustard (*Brassica hirta)*	189 ng/g DW	18.9 µg/100 g DW	
	Almonds (*Prunus amydalus)*	39 ng/g DW	3.9 µg/100 g DW	
	Broccoli (*Brassica oleraceae* L.)	0.41 ng/g DW	41 ng/100 g DW	
Legumes	Kidney beans (*Phaseolus vulgaris* L.)	529.1 ng/g DW	52.9 µg/100 g DW	For overview see [7]
	Soya bean (Glycine max L.)	1.89 ng/g DW	189 ng/100 g DW	
Nuts	Pistachio (*Pistacia vera* L. cv. Ahmad Aghaei)	233,000 ng/g DW	23.3 µg/100 g DW	For overview see [7]
	Walnut (*Juglans regia* L. cv. Hartley)	1.77 ng/g FW	177 ng/100 g FW	
Fruits	Cherry (*Prunus avium* L. cv. Hongdeng)	10–20 ng/g FW	1–2 µg/100 g FW	For overview see [7]
	Strawberry (*Fragaria ananassa* L. cv. Primoris)	8.5 ng/g FW	8.5 µg/100 g FW	
	Apple (*Malus domestica Borkh*. cv. Red Fuji)	5 ng/g FW	500 ng/100 g FW	
	Mango (*Mangifera indica)*	2.401 ng/g DW	240.1 ng/100 g DW	[16]
	Mulberry (*Morus alba)*	1.739 ng/g DW	173.9 ng/100 g DW	
	Pineapple (*Ananas comosus)*	1.693 ng/g DW	169.3 ng/100 g DW	
Animal products	Cow milk	14.45 pg/mL	1.4 ng/100 mL	For overview see [7]
	Salmon	3.7 ng/g	370 ng/100 g	
	Pork	2.5 ng/g	250 ng/100 g	
	Egg (raw, whole)	1.54 ng/g	154 ng/100 g	
	Probiotic yogurt	0.126 ng/mL	12.6 ng/100 mL	[8]

*DW* dry weight, *FW* fresh weight

The aim of the present study was to determine whether an oral supplementation of low doses (= “dietary doses”) of melatonin (50 ng/kg BW/day) attenuates the development of ALD in mice and to determine underlying molecular mechanisms.

## Materials and methods

### Animals and treatment

All animal protocols were approved by the local Institutional Animal Care and Use Committee (Austrian Federal Ministry of Education, Science and Research, 2022–0.039.040). Female C57BL/6J mice (6–8 weeks) were obtained from Janvier SAS France and housed in a SPF facility accredited by the Association for Assessment and Accreditation of Laboratory Animal Care. After adapting mice to the animal facility and the intake of the liquid Lieber DeCarli control diet (IPS Product Supplies Ltd, Alfreton, UK), mice were randomized to either consume the control Lieber DeCarli diet (C) or a Lieber DeCarli diet enriched with ethanol (EtOH) as described before [17]. In brief, mice (4 groups; *n* = 6–8/group) were adapted to the ethanol-enriched Lieber DeCarli liquid diet for 12 days starting from 2% ethanol and increasing the concentration by 1% every third day. Maximum ethanol concentration (5%) was given for 20 days (Online Resource 1). Additionally, liquid diets of one ethanol and one control group were supplemented with 50 ng melatonin/kg BW/day (Sigma-Aldrich, Germany). Doses of melatonin were based on a “physiological” nutrition-based intake in humans consuming a typical plant-rich diet (Table 1, [7]). It has been shown that different foods (e.g., fruits, vegetables) [7, 8] and beverages (e.g., wine, beer, coffee) [13–15] commonly found in Westernized countries contain marked amounts of melatonin. Mice had ad libitum access to water. Consumption of diet and melatonin was controlled and adapted daily; body weight was assessed weekly. At the end of the experiment, mice were anesthetized with 100 mg ketamine/16 mg xylazine/kg BW (i.p.) and killed by cervical dislocation. Blood was collected from the portal vein; liver and intestine were collected and either fixed in neutral-buffered formalin or snap-frozen and stored at − 80 °C.

### Everted gut sac experiment

Further investigations were performed using the ex vivo everted gut sac model as described before [18]. Everted gut sacs were built from small intestine of naïve female C57BL/6J mice (*n* = 5/treatment). In a first experiment, tissue sacs were incubated with ± 0.5‰ (0.5 g/L) ethanol and ± 1 mM melatonin in 1 × Krebs–Henseleit-bicarbonate-buffer containing 0.2% bovine serum albumin (KRH) at 37 °C for 55 min followed by a 5 min incubation with 0.1% xylose. In a second set of experiment, tissue sacs were incubated with ± 0.5‰ ethanol and ± 1 mM melatonin in KRH at 37 °C for 55 min followed by the 5 min incubation with 0.1% xylose. Additionally, some of the tissue sacs challenged with ethanol and melatonin were concomitantly incubated with ± 10 µM compound C (CompC; Sigma-Aldrich). Intestinal tissue was snap frozen for further analysis.

### Statistics

For statistical analysis, the program PRISM (Version 7.03, GraphPad Software, Boston, MA, USA) was used. Data were tested for outliers using the Grubb’s outlier test. Normality of distribution was assessed using the Shapiro–Wilk normality test. For testing equality of group variances, Brown-Forsythe test was performed. To analyze data from the in vivo study, a two-way analysis of variance was performed. A one-way analysis of variance was used to compare the different treatments in the ex vivo everted gut sac model. Statistical differences were defined as *p* ≤ 0.05.

All further methods used are described in Online Resource 2.

## Results

### Effect of an oral low-dose melatonin supplementation on markers of liver damage and inflammation

**Fig. 1:**
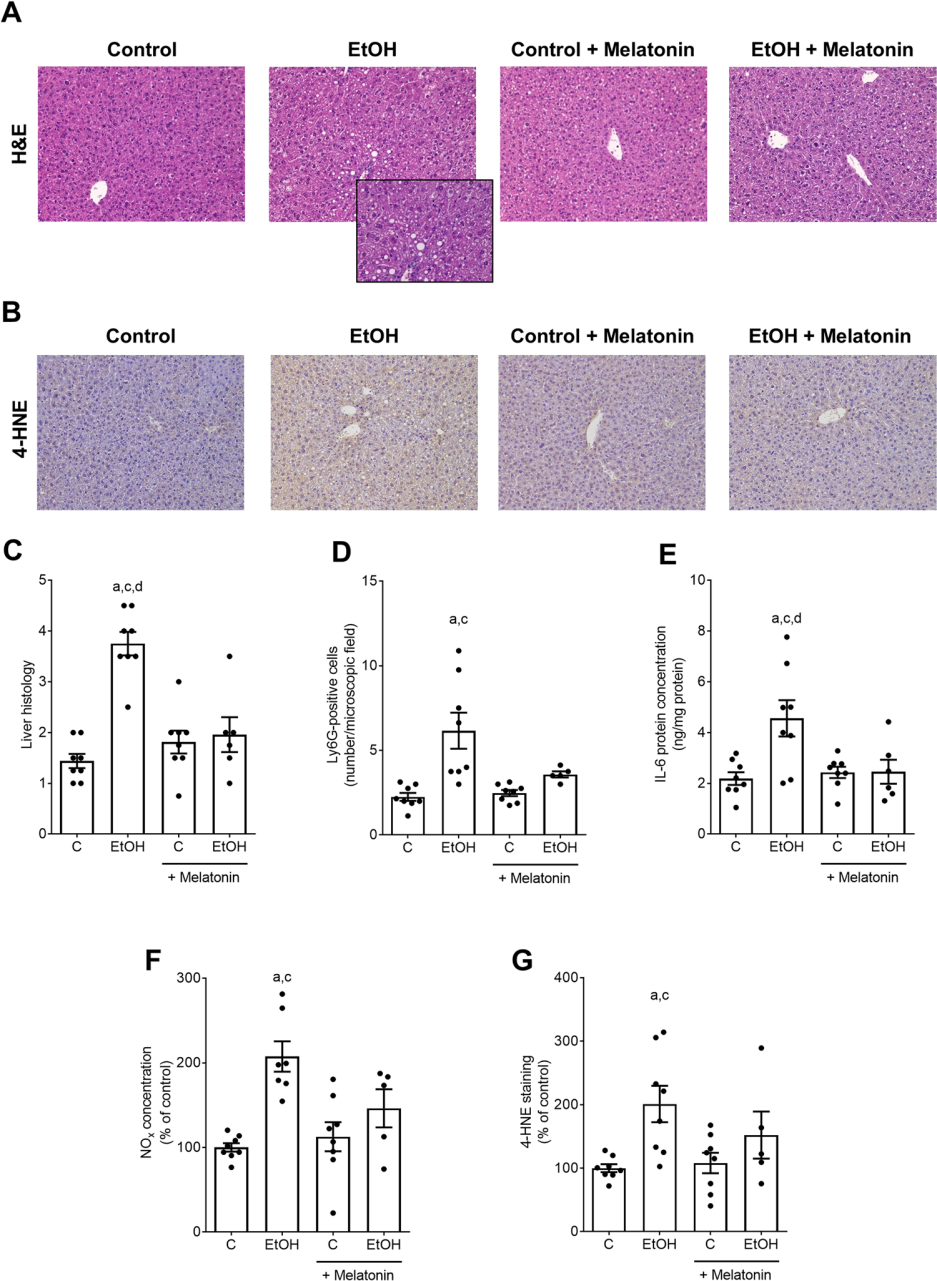
Effects of an oral supplementation of melatonin on markers of liver damage in mice fed a liquid alcohol containing or control diet. Representative pictures of hematoxylin & eosin (H&E) staining (magnification 200 × and 400 x) **A** and 4-HNE protein adduct staining (magnification 200 x) in liver tissue **B**, scoring of liver sections **C** and number of Ly6G-positive cells in liver sections **D**. IL-6 protein concentration **E**, NOx concentration in liver tissue **F** as well as evaluation of 4-HNE protein adduct staining in liver **G**. Data are presented as mean ± SEM; ^a^ *p* ≤ 0.05 compared to C; ^c^ *p* ≤ 0.05 compared to C + Melatonin; ^d^ *p* ≤ 0.05 compared to EtOH + Melatonin. 4-HNE: 4-hydroxynonenal protein adducts; C: control diet, EtOH: ethanol-enriched Lieber DeCarli liquid diet, IL-6: interleukin 6; Ly6G, lymphocyte antigen 6 complex locus G6D; NOx: nitrite

As animals were pair-fed, caloric intake was similar whereas body weight was significantly lower in mice fed the alcohol-enriched Lieber DeCarli diet fortified with melatonin. Absolute body weight gain was significantly lower in both ethanol-fed groups compared to controls Table 2. Alcohol intake of the two alcohol-fed groups was similar Table 2. Absolute liver weight was only significantly higher in mice fed the alcohol-enriched Lieber DeCarli diet while liver-to-body weight ratio was significantly higher in both alcohol-fed groups compared to controls Table 2. In line with previous studies of others [19], mice fed the alcohol-enriched Lieber DeCarli diet showed mild steatosis with early signs of hepatic inflammation Fig. 1A, C. In mice concomitantly treated with melatonin while being exposed to alcohol, the development of steatosis and inflammation was significantly attenuated compared to mice only fed ethanol. Indeed, both, liver fat accumulation and inflammation were almost at the level of controls. Moreover, number of lymphocyte antigen 6 complex locus G6D (Ly6G)-positive cells were significantly higher in mice fed plain ethanol compared to both control groups Fig. 1D. Differences alike were not found when comparing number of Ly6G-positive cells in livers of ethanol-fed mice concomitantly treated with melatonin with all other groups. Protein levels of interleukin 6 (IL-6) were significantly higher in mice fed the alcohol-enriched Lieber DeCarli diet than in all other groups. In livers of ethanol-fed mice concomitantly treated with melatonin, IL-6 protein concentrations were at the level of controls Fig. 1E. Concentration of nitrite (NOx) and 4-hydroxynonenal protein adducts (4-HNE) were also significantly higher in mice fed the alcohol-enriched Lieber DeCarli diet than in both control groups. Similar differences were not found when NOx concentrations and 4-HNE protein adduct levels in liver tissue were compared between controls and ethanol-fed mice treated with melatonin Fig. 1B, F, G. Activity of alanine aminotransferase (ALT) in plasma was significantly higher in both ethanol-fed groups irrespective of additional treatments compared to controls Table 2.

**Table 2:** Effect of an oral supplementation of melatonin on caloric intake, body, and liver weight as well as liver damage in mice fed a liquid alcohol containing or control diet

	Diet and treatment groups			
	C	EtOH	C + Melatonin	EtOH + Melatonin
Caloric intake (kcal/g BW)	0.38 ± 0.0	0.35 ± 0.0	0.38 ± 0.0	0.36 ± 0.0
Ethanol intake (g/day)	-	0.36 ± 0.01	-	0.37 ± 0.02
Body weight (g)	23.4 ± 0.7	21.9 ± 0.4	23.3 ± 0.3	20.5 ± 0.2 ^a,c^
Absolute body weight gain (g)	5.2 ± 0.4	1.7 ± 0.4 ^a,c^	5.5 ± 0.5	1.2 ± 0.2 ^a,c^
Liver weight (g)	1.01 ± 0.0	1.17 ± 0.0 ^a,c^	1.01 ± 0.0	1.14 ± 0.1
Liver-to-body weight ratio (%)	4.3 ± 0.1	5.4 ± 0.1 ^a,c^	4.3 ± 0.1	5.5 ± 0.2 ^a,c^
ALT (U/l)	17.8 ± 1.5	46.0 ± 2.7 ^a,c^	20.0 ± 2.0	50.3 ± 10.5 ^a,c^

Data are shown as means ± SEM, *n*= 6–8, *ALT* alanine aminotransferase, *C* control diet, *EtOH* 5% ethanol-enriched Lieber DeCarli liquid diet

^a^ *p*≤ 0.05compared to C; ^c^*p*≤ 0.05compared to C + Melatonin

### Effect of an oral low-dose melatonin supplementation on markers of intestinal barrier function, NOx concentration and phospho-5′-AMP-activated protein kinase alpha (pAMPKα) in small intestine

**Fig. 2:**
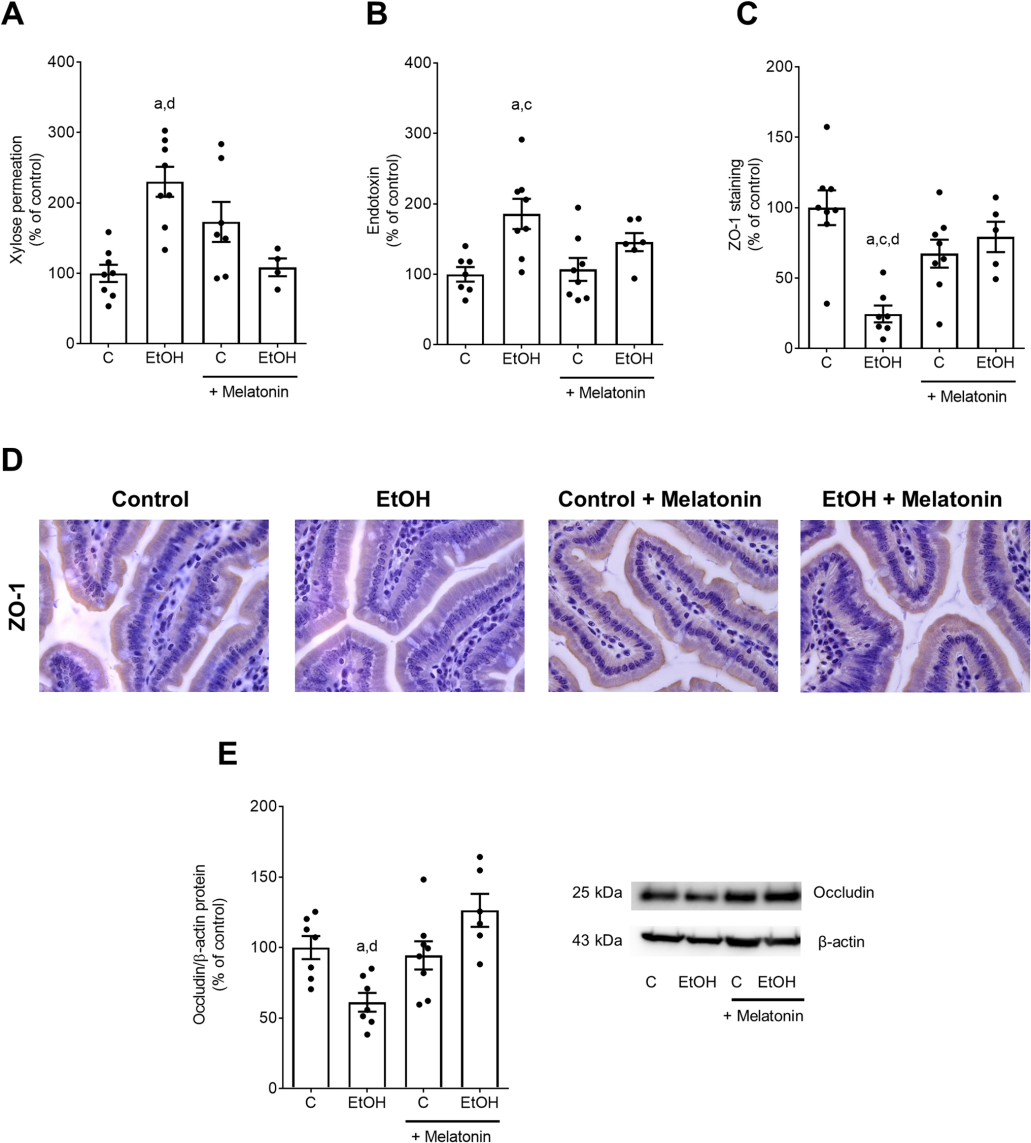
Effects of an oral supplementation of melatonin on markers of intestinal permeability in mice fed a liquid alcohol containing or control diet. Xylose permeation assessed in everted small intestinal tissue sacs **A**, bacterial endotoxin concentration in portal plasma **B**, evaluation **C** and representative pictures of ZO-1 staining **D** and protein levels and representative blots of occludin in relation to β-actin in small intestine **E**. Data are presented as mean ± SEM; ^a^*p* ≤ 0.05 compared to C; ^c^*p* ≤ 0.05 compared to C + Melatonin; ^d^*p* ≤ 0.05 compared to EtOH + Melatonin. C: control diet, EtOH: ethanol-enriched Lieber DeCarli liquid diet, ZO-1: zonula occludens 1

**Fig. 3:**
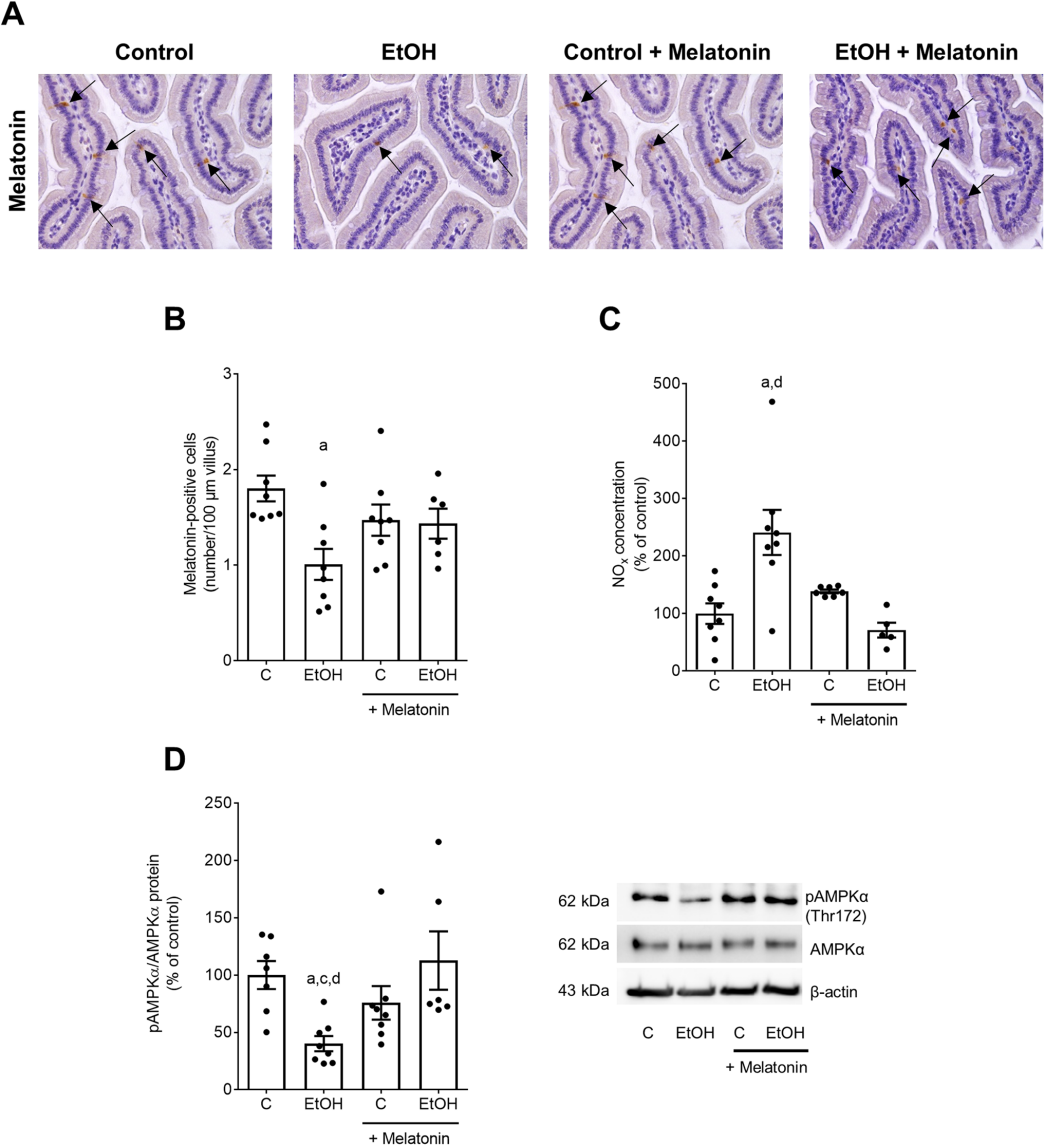
Effects of an oral supplementation of melatonin on NOx concentration and phospho-5′-AMP-activated protein kinase alpha (pAMPKα) in small intestine in mice fed a liquid alcohol containing or control diet. Representative pictures **A** and evaluation of melatonin-positive cells per 100 µm villus in small intestine **B**. NOx concentration **C** as well as protein levels of pAMPKα protein in relation to AMPKα in small intestine and representative blots of pAMPKα, AMPKα and β-actin **D**. Data are presented as mean ± SEM; ^a^*p* ≤ 0.05 compared to C; ^c^*p* ≤ 0.05 compared to C + Melatonin; ^d^*p* ≤ 0.05 compared to EtOH + Melatonin. Black arrows represent melatonin-positive cells in small intestine. AMPK: 5′-AMP-activated protein kinase; C: control diet, EtOH: ethanol-enriched Lieber DeCarli liquid diet, NOx, nitrite; pAMPK: phospho-AMPK

As it has been shown before that melatonin may affect intestinal barrier function [12] and that acute and chronic intake of ethanol may damage intestinal barrier function especially in upper parts of the small intestine [20], we next determined markers of intestinal barrier function. Intestinal permeability was assessed by incubating everted gut sacs with xylose obtained from animals at the end of the feeding trial. Xylose permeation was significantly higher in ethanol-fed mice than in controls and ethanol-fed mice treated with melatonin Fig. 2A. Endotoxin concentration in portal plasma were significantly higher in ethanol-fed animals compared to controls. Differences alike were not found when comparing mice fed the ethanol-diet enriched with melatonin and controls Fig. 2B. Zonula occludens 1 (ZO-1) protein levels in small intestine were significantly lower in ethanol-fed mice compared not only to control groups but also to ethanol-fed mice treated with melatonin. ZO-1 protein levels were at the level of controls in ethanol-fed mice treated with melatonin Fig. 2C, D. Occludin protein concentration in small intestine was significantly lower in ethanol-fed animals than in controls and ethanol-fed mice treated with melatonin Fig. 2E. Numbers of melatonin-positive cells in small intestine were significantly lower in mice fed the ethanol-enriched diet compared to their respective control group. In small intestine of ethanol-fed mice concomitantly treated with melatonin, a number of melatonin-positive cells were at the level of controls Fig. 3A, B. Expressions of melatonin receptor 1 (*Mtr1*) mRNA neither differed in liver nor in small intestine among groups. Expression of *Mtr2* mRNA was below the level of detection in either tissue and groups Table 3. Moreover, the significant increase in NOx levels in small intestinal tissue in mice fed the ethanol-diet was significantly attenuated in mice fed the ethanol-diet supplemented with melatonin Fig. 3C. NOx levels in small intestinal tissue of mice fed the ethanol-enriched Lieber DeCarli diet supplemented with melatonin were at the level of controls Fig. 3C. Furthermore, the ratio of pAMPKα to AMPKα was significantly lower in small intestinal tissue of ethanol-fed mice compared to all other groups while not differing between ethanol-fed mice concomitantly treated with melatonin Fig. 3D.

**Table 3:** Effect of an oral supplementation of melatonin on mRNA expression of melatonin receptors in livers and small intestine in mice fed a liquid alcohol containing or control diet

	Diet and treatment groups			
	C	EtOH	C + Melatonin	EtOH + Melatonin
In liver				
*** Mtr1*** mRNA expression^#^	100.0 ± 17.2	152.5 ± 23.2	81.3 ± 14.4	156.1 ± 37.2
*** Mtr2*** mRNA expression^#^	ND	ND	ND	ND
In small intestine				
*** Mtr1*** mRNA expression^#^	100.0 ± 20.7	73.0 ± 17.1	84.2 ± 19.4	49.4 ± 6.1
*** Mtr2*** mRNA expression^#^	ND	ND	ND	ND

Data are shown as means ± SEM, *n*=6–8. ^#^% of control

*C* control diet, *EtOH* 5% ethanol-enriched Lieber DeCarli liquid diet, *Mtr* melatonin receptor, *ND* not detectable

### Effect of melatonin on intestinal permeability and AMPK phosphorylation in everted gut sacs challenged with 0.5‰ ethanol ex vivo

**Fig. 4:**
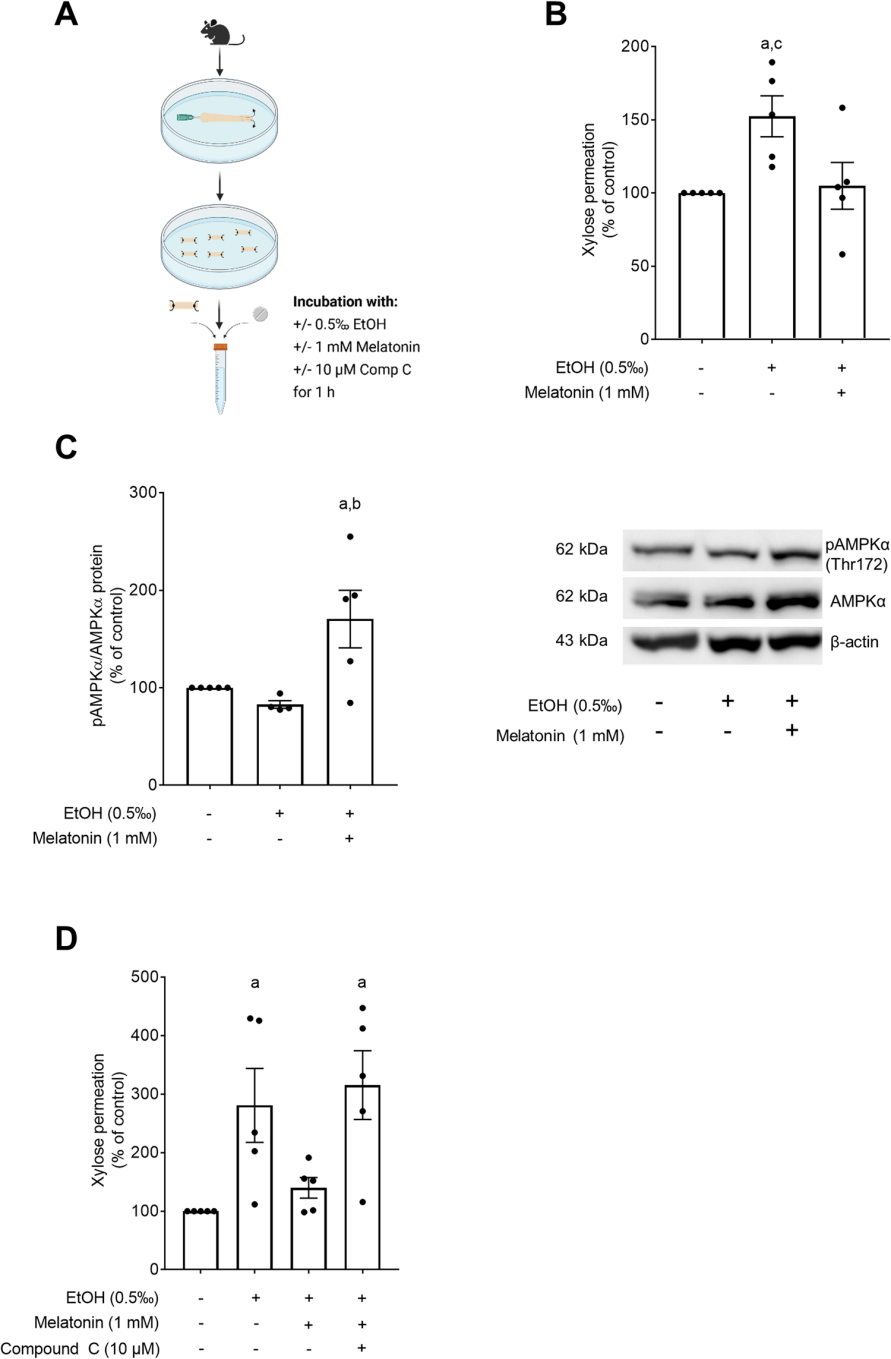
Effects of melatonin on EtOH-induced intestinal barrier dysfunction in ex vivo small intestinal tissue everted gut sacs. Experimental setup of the ex vivo everted gut sac model **A**, xylose permeation in everted small intestinal tissue sacs treated with ± 0.5‰ EtOH and ± 1 mM melatonin **B**, relative levels of pAMPKα protein in relation to AMPKα protein and representative blots of pAMPKα, AMPKα and β-actin **C** as well as xylose permeation in everted small intestinal tissue sacs treated with ± 0.5‰ EtOH, ± 1 mM melatonin and ± 10 µM AMPK inhibitor CompC **D**. Data are presented as mean ± SEM; ^a^*p*≤ 0.05 compared to C; ^b^*p* ≤ 0.05 compared to EtOH; ^c^*p* ≤ 0.05 compared to EtOH + Melatonin. AMPK: 5′-AMP-activated protein kinase, C: control, EtOH: 0.5‰ EtOH, CompC: compound C, pAMPK: phospho-AMPK

In line with the findings in in vivo experiments, intestinal permeability was significantly higher in everted gut sacs challenged with 0.5‰ ethanol compared to control and tissue sacs exposed to ethanol in the presence of melatonin Fig. 4B. The protective effect of melatonin was related to a significant increase in phosphorylation of AMPK in ethanol-treated tissue sacs concomitantly exposed to melatonin Fig. 4C. Moreover, when ethanol-challenged everted gut sacs were concomitantly exposed to melatonin in the presence of the AMPK inhibitor compound C, the protective effects of melatonin on the ethanol-related increase in intestinal permeability were almost completely abolished Fig. 4D.

## Discussion

Alcohol abstinence is the therapy of choice in the treatment of ALD. However, especially when being related to alcohol use disorder (AUD), relapse rates are often high [21]. Only very few clinical trials have been conducted employing melatonin in patients with AUD. These studies focused mainly on its effect on sleep disturbances and found divergent results. Gendy et al. showed no differences between the melatonin treatment group and a placebo group [22]. Another study, employing a melatonin agonist, showed an improved sleep quality in alcohol-dependent patients; however, this study was not placebo-controlled [23]. Supplementation of melatonin at high doses has been reported before to alleviate some of the effects of ethanol on the liver, and herein, especially inflammatory alterations; however the latter was found in rodents [24]. Here, using the Lieber DeCarli ethanol feeding model in female mice, we assessed the effects of an oral supplementation of “physiological” doses of melatonin on the development of ALD. It has been shown that female mice were more susceptible to alcohol-related liver damage ([25]; for overview [26]). Besides differences in body composition, e.g., high fat mass in female mice and ethanol metabolism (ADH isoenzymes) [27], estrogen and growth factors have been shown to be critical in these sex-specific differences. Studies have shown that estrogen may enhance liver inflammation, oxidative stress, and Kupffer cell sensitivity to endotoxins thereby further adding to the vulnerability to ALD in female mice [28]. In the present study, the chronic oral supplementation of “physiological” doses of melatonin diminished the development of alcohol-related liver damage. Specifically, in livers of mice fed the ethanol containing diet enriched with melatonin, signs of early inflammation like an increased number of Ly6G-positive cells and inflammatory foci were markedly diminished being also related to lesser fat accumulation. Moreover, the increases in NO_x_, 4-HNE protein adducts, and IL-6 protein found in livers of mice fed the ethanol-diet were attenuated suggesting that animals were protected from oxidative stress shown before to be critical in the development of ALD [29]. These findings are in line with those of others using pharmacological doses, e.g., 10–50 mg/kg (200–1000 fold the dose used in the present study) [9]. Reports of the effect of a supplementation of high doses of melatonin on transaminase activity in serum of rodents in ALD models or in humans with ALD are contradictory [30]. Specifically, no effects or a lowering effect were found. In contrast, the treatment with high doses of melatonin regardless if administered orally or i.p. has been related with a reduction of proinflammatory cytokine concentration (e.g., IL-6) and NOx levels as well as markers of reactive oxygen species in liver tissue in settings of ALD [9]. The lack of responsiveness to the supplementation of melatonin regarding ALT levels but also liver-to-body weight ratio found in the present study could be related to the fact that data varied considerably in the ethanol groups. Moreover, studies of others suggested before that ALT activity in blood may be more indicative for accumulation of fat in the liver than inflammation [31, 32]. In the present study, changes in liver fat content were less pronounced than those in inflammation. Also, a meta-analysis has reported that melatonin can improve liver indices but has no significant effect on ALT levels in MASLD patients [33]. It has been discussed before that an induction of IL-6 may be part of the host defense strategy [34]. The lower protein levels of IL-6 found in the present study may suggest that mice were generally protected from the development of ALD, and therefore, the necessity of an induction of host defense strategies was lower than in mice fed the plain ethanol-diet. This needs to be determined in future studies. Taken together, these results add further weight to the hypothesis that a supplementation of melatonin in “physiological” doses may have beneficial effects on the development of early stages of ALD in rodents. However, as in the present study, the liquid diet and therefore also melatonin was readily available 24 h a day; further studies are needed to determine effects of circadian rhythm. Also, it remains to be determined if doses like the one used in the present study also have an effect on later stages of the disease and if protective effects are also found in humans.

### Melatonin attenuates alcohol-induced small intestinal barrier dysfunction

Studies have repeatedly shown that alcohol may not only affect liver function through its metabolism and the related shift in the ratio of NADH + H^+^ and NAD^+^ but that alterations of intestinal microbiota composition and intestinal barrier function may be critical in the development of ALD, too [35]. Subsequently, this has been shown to result in an increased translocation of bacterial endotoxin and activation of TLR4 signaling cascades in liver tissue [35]. Studies further suggest that attenuating the loss of intestinal barrier function or blocking the endotoxin-TLR4 signaling cascade in liver tissue is related to a protection from the development of ALD [36]. In the present study, the beneficial effects of fortifying the liquid alcohol diet with low doses of melatonin were related with a protection against the loss of melatonin and the increase of intestinal permeability in small intestinal tissue. It has been shown that levels of circulating melatonin are lower in individuals with AUD and that this is related to higher intestinal permeability [37]. Interestingly, in the present study, the numbers of melatonin-positive cells were at the level of controls in small intestine of mice treated with melatonin. These data suggest that melatonin supplementation may have induced a feedback inhibition mechanism of endogenous melatonin synthesis, maybe specifically in the gastrointestinal tract—known as a major site of endogenous melatonin synthesis [6]. Somewhat in line with the present study, others reported that melatonin at pharmacological doses may attenuate the development of MASLD through mechanisms involving an improvement of intestinal barrier function by involving an attenuation of the loss of tight junctions [9]. Results of in situ studies suggest that through its receptor melatonin may decrease paracellular permeability [38]. It also has been shown before that an acute and chronic exposure to melatonin may reduce increases in duodenal permeability in rats induced through an acute ethanol exposure [12] being related to an upregulation of *Mtr1*, *Mtr2*, and *claudin-2* mRNA expression [39]. Interestingly, mRNA expression of *occludin* and *ZO-1* were unchanged by the melatonin treatment [39]. The latter results somewhat contrast the results of the present study where ZO-1 protein was significantly reduced in mice fed the ethanol-diet, while in ethanol-fed mice concomitantly receiving melatonin, ZO-1 protein levels were at the level of controls. Studies have shown that tight junction proteins may also be regulated post-translationally [40]. Also, doses and experimental setup were markedly different between the present study and that of others [39]. Moreover, *Mtr1* mRNA expressions in liver and small intestine did not differ within groups while *Mtr2* mRNA was below the detection level. This latter finding contrasts recent findings of others detecting protein of both melatonin receptors in ileal mucosa of mice [41]. Differences between our study and that of others could be related to the section of the small intestine used (the upper/middle part in the present study vs. ileum). Studies showed that expression levels of *Mtr1* and *2* may vary between species, tissues, and detection method [42]. It has been suggested before that in mice binding of 2-[^125^I]iodomelatonin (melatonin agonist) in small intestine was lower than in other species [43]. Still, in line with the findings for *Mtr1* mRNA expression in the present study, others showed no effects of melatonin supplementation on Mtr protein levels in small intestine [41]. Results of a recent study of our own group employing mouse models of MASLD but also studies of others exposing mice to alcohol suggest that alterations of AMPK activity and a disbalance of NO-homeostasis may be critical in the development of intestinal barrier dysfunction ([44]; for overview [45–47]). Moreover, studies suggest that melatonin may affect blood–brain barrier through activating AMPK [48]. In the present study, the protection against intestinal barrier dysfunction in ethanol-fed mice concomitantly treated with melatonin was related to an attenuation of the loss of AMPK activity as determined by assessing phosphorylation of Thr172 in AMPKα shown before to be critical in the regulation of AMPK [49] and an increase of NOx. Further suggesting that melatonin affected intestinal barrier function through altering AMPK activity the challenge of everted gut tissue sacs with ethanol in the presence of melatonin was related to an increase in phosphorylated AMPK. Moreover, pre-treating everted tissue sacs with compound C attenuated the protective effects of melatonin on intestinal barrier dysfunction. When interpreting the results of the everted sac model, it has to be considered that this model by no means mimics the situation present in the gut in a setting of chronic alcohol intake in vivo.

Our results suggest that low doses of melatonin might be sufficient to attenuate the increase in intestinal permeability in small intestine and that is potentially through stabilization of AMPK activity. Further studies are needed to determine how mechanisms underlying the AMPK pathway interact with the synthesis of NOx and tight junctions. Our results by no means preclude that melatonin also exhibits anti-oxidant, anti-inflammatory, and anti-fibrotic properties in hepatocytes and non-parenchymal liver cells which may affect the development of ALD [50]. If effects alike are also found when an alcohol-related damage of intestinal barrier in small intestine is already present and if this is also relevant in humans with high, regular alcohol consumption remains to be determined.

## Conclusion

Results of the present study suggest that an oral supplementation of ‘physiological’ doses of melatonin which could be achieved through diet in humans may diminish the development of ALD. Our data also indicates that the protective effects of melatonin on the development of ALD in mice may be related to its effects on ethanol-induced intestinal barrier dysfunction in small intestine. Herein, our data suggest that melatonin may alleviate the ethanol-induced increases in NOx formation and the loss of AMPK activity in small intestinal tissue. However, further studies are needed to delineate molecular mechanisms underlying the interaction of melatonin with AMPKα and the related effects on intestinal barrier function. Furthermore, it remains to be determined if a diet rich in melatonin derived through natural sources has similar protective effects and if these are also found in humans, especially in settings of chronic elevated alcohol consumption.

## Supplementary Information

Below is the link to the electronic supplementary material.

ESM1 (PDF 69.7 KB)

ESM2 (PDF 121 KB)

ESM3 (PDF 40.7 KB)

ESM4 (PDF 91.3 KB)

ESM5 (PDF 114 KB)

ESM6 (PDF 125 KB)

## Data Availability

Data can be made available upon reasonable request.

## Abbreviations

4-HNE: 4-Hydroxynonenal protein adducts
ALD: Alcohol-related liver disease
ALT: Alanine aminotransferase
AMPK: 5′-AMP-activated protein kinase
AUD: Alcohol use disorder
C: Control Lieber DeCarli diet
EtOH: Ethanol-enriched Lieber DeCarli diet
H&E: Hematoxylin and eosin
IL-6: Interleukin 6
KRH buffer: 1X Krebs–Henseleit-bicarbonate-buffer containing 0.2% bovine serum albumin
Ly6G: Lymphocyte antigen 6 complex locus G6D
MASLD: Metabolic dysfunction-associated steatotic liver disease
Mtr: Melatonin receptor
NOx: Nitrite
ZO-1: Zonula occludens 1
