# Supplementary material for: Oral supplementation of melatonin attenuates the onset of alcohol-related liver disease
Source: J Mol Med (Berl). 2025 Aug 7;103(10):1219–30. doi: 10.1007/s00109-025-02583-4 (PMC12449381; doi:10.1007/s00109-025-02583-4)
Supplement: Supplementary file 2 — (PDF 121 KB) [file 109_2025_2583_MOESM2_ESM.pdf]

Oral Supplementation of Melatonin Attenuates the Onset of Alcohol-Related Liver Disease

Franziska Kromm, Anja Baumann, Victor Sánchez, Annette Brandt, Raphaela Staltner, Ina Bergheim*

* **Corresponding author:** Ina Bergheim, Ph.D.
University of Vienna
Department of Nutritional Sciences
Molecular Nutritional Science
Josef-Holaubek-Platz 2 (UZA II)
A-1090 Vienna
E-Mail: ina.bergheim@univie.ac.at

Online Resource 2. Further methods used in the present study

Determination of small intestinal permeability *ex vivo*

To determine intestinal permeability, small intestinal tissue from mice of the *in vivo* trial as well as from naïve mice was everted, and tissue sacs were formed which were then exposed to 0.1% xylose for 5 min at 37°C as detailed before [1]. Concentration of D-xylose was measured using phloroglucinol as previously described [2].

Evaluation of liver damage

Paraffin-embedded sections of liver (4 µm) were stained with hematoxylin and eosin (Sigma-Aldrich) and scored using the Nanji score [3]. Number of lymphocyte antigen 6 complex locus G6D (Ly6G)-positive cells were stained with a monoclonal antibody (anti-Ly6G, abcam Cambridge, UK) as previously described [4]. Activity of alanine aminotransferase (ALT) was measured as a routine laboratory parameter at the University of Veterinary Medicine Vienna.

ELISA

Interleukin 6 (IL-6) protein levels in liver tissue were assessed using a commercially available ELISA Kit (DuoSet ELISA Kits, R&D Systems, Minneapolis, Minnesota, USA).

Nitrite (NOx)

Nitrite (NOx) levels in liver and small intestine were determined using a commercially available Griess reagent kit according to the manufacturer (Promega, Madison, Wisconsin, USA) and normalized to protein as detailed before [1].

Endotoxin measurement

Bacterial endotoxin levels in plasma were measured with a commercially available Limulus amebocyte lysate assay (Charles River Laboratories, Sulzfeld, Germany) as described in detail before [5]. Recovery rates ranged from ~88-120%.

Immunohistochemical staining

Paraffin-embedded sections (4 µm) of liver and small intestine were used for immunohistochemical staining. Sections of liver were incubated with 4-hydroxynonenal protein adducts (4-HNE) using polyclonal antibody (AG Scientific, San Diego, California, USA) as described before [6]. Sections of small intestine were stained with a primary antibody for melatonin (Abbexa, Cambridge, UK) as described by others before [7]. In brief,

deparaffinized sections were treated with 3% H_2O_2 and citrate buffer before the incubation with a primary antibody for melatonin and a respective secondary antibody. Furthermore, sections of small intestine were incubated with primary antibody for the tight junction protein zonula occludens 1 (ZO-1; Invitrogen, Waltham, Massachusetts, USA) as described before [8]. The staining of ZO-1 and 4-HNE was analyzed using a software integrated with a camera (Leica DM6B & DMC4500, Leica, Wetzlar, Germany) [6,8]. To determine number of melatonin positive cells, cells stained positively were counted in 20 villi (magnification 400 x) per section. Length of villi used for counting was measured, and results are shown in melatonin-positive cells per 100 µm villi length.

Western Blot

Protein of small intestine of the animal trial and *ex vivo* everted sac experiments was isolated with Trizol (VWR, Vienna, Austria), separated on 12% SDS polyacrylamide gels and transferred to polyvinylidene difluoride membranes (Bio-Rad Laboratories, Hercules, California, USA) as detailed before [9]. Membranes were incubated with primary antibodies (5' AMP-activated protein kinase (AMPKα); abcam, phospho-AMPKα (Thr172; pAMPK); Cell Signaling, Leiden, Netherlands or occludin; Thermo Fisher Scientific, Waltham, Massachusetts, USA) as well as β-actin (Santa Cruz Biotechnology, Inc., Heidelberg, Germany) over night at 4°C and with respective HRP-linked secondary antibodies. Visualization of protein bands was performed using Super Signal West Dura kit (Thermo Fisher Scientific, Waltham, Massachusetts, USA). Densitometrical analysis of detected bands was accomplished using the ChemiDoc XRS System with Image Lab software (Image Lab 6.1, Bio-Rad Laboratories).

RNA isolation and real-time PCR

RNA was isolated from both liver and small intestine using the commercially available TRItidy G (Applichem, Darmstadt, Germany) and was then reverse transcribed using a reverse-transcription system (Promega GmbH, Mannheim, Germany). Real-time PCR was performed as previously described [10]. Expressions of respective genes were normalized to 18S. Primer sequences are shown in Online Resource 3.

References

1. Rajcic D, Baumann A, Hernandez-Arriaga A, et al (2021) Citrulline supplementation attenuates the development of non-alcoholic steatohepatitis in female mice through mechanisms involving intestinal arginase. Redox Biol 41101879. https:\\doi.org\10.1016/j.redox.2021.101879

2. Eberts TJ, Sample RH, Glick MR, Ellis GH (1979) A simplified, colorimetric micromethod for xylose in serum or urine, with phloroglucinol. Clin Chem 25(8):1440-1443.

3. Nanji AA, Tsukamoto H, French SW (1989) Relationship between fatty liver and subsequent development of necrosis, inflammation and fibrosis in experimental alcoholic liver disease. Exp Mol Pathol 51(2):141-148. https:\\doi.org\10.1016/0014-4800(89)90014-2

4. Sanchez V, Baumann A, Brandt A, Wodak MF, Staltner R, Bergheim I (2024) Oral Supplementation of Phosphatidylcholine Attenuates the Onset of a Diet-Induced Metabolic Dysfunction-Associated Steatohepatitis in Female C57BL/6J Mice. Cell Mol Gastroenterol Hepatol 17(5):785-800. https:\\doi.org\10.1016/j.jcmgh.2024.01.009

5. Nier A, Engstler AJ, Maier IB, Bergheim I (2017) Markers of intestinal permeability are already altered in early stages of non-alcoholic fatty liver disease: Studies in children. PLoS One 12(9):e0183282. https:\\doi.org\10.1371/journal.pone.0183282

6. Jin CJ, Sellmann C, Engstler AJ, Ziegenhardt D, Bergheim I (2015) Supplementation of sodium butyrate protects mice from the development of non-alcoholic steatohepatitis (NASH). Br J Nutr 114(11):1745-1755. https:\\doi.org\10.1017/S0007114515003621

7. Wang B, Zhu S, Liu Z, et al (2020) Increased Expression of Colonic Mucosal Melatonin in Patients with Irritable Bowel Syndrome Correlated with Gut Dysbiosis. Genomics Proteomics Bioinformatics 18(6):708-720. https:\\doi.org\10.1016/j.gpb.2020.06.013

8. Brandt A, Hernandez-Arriaga A, Kehm R, et al (2019) Metformin attenuates the onset of non-alcoholic fatty liver disease and affects intestinal microbiota and barrier in small intestine. Sci Rep 9(1)6668. https:\\doi.org\10.1038/s41598-019-43228-0

9. Wagnerberger S, Spruss A, Kanuri G, et al (2013) Lactobacillus casei Shirota protects from fructose-induced liver steatosis: a mouse model. J Nutr Biochem 24(3):531-538. https:\\doi.org\10.1016/j.jnutbio.2012.01.014

10. Baumann A, Burger K, Brandt A, et al (2022) GW9662, a peroxisome proliferator-activated receptor gamma antagonist, attenuates the development of non-alcoholic fatty liver disease. Metabolism 133:155233. https:\\doi.org\10.1016/j.metabol.2022.155233
