# Supplementary material for: Oral supplementation of melatonin attenuates the onset of alcohol-related liver disease
Source: J Mol Med (Berl). 2025 Aug 7;103(10):1219–30. doi: 10.1007/s00109-025-02583-4 (PMC12449381; doi:10.1007/s00109-025-02583-4)
Supplement: Supplementary file 3 — (PDF 40.7 KB) [file 109_2025_2583_MOESM3_ESM.pdf]

Oral Supplementation of Melatonin Attenuates the Onset of Alcohol-Related Liver Disease

Franziska Kromm, Anja Baumann, Victor Sánchez, Annette Brandt, Raphaela Staltner, Ina Bergheim*

* **Corresponding author:** Ina Bergheim, Ph.D.
University of Vienna
Department of Nutritional Sciences
Molecular Nutritional Science
Josef-Holaubek-Platz 2 (UZA II)
A-1090 Vienna
E-Mail: ina.bergheim@univie.ac.at

Online Resource 3. Primer sequences used for real-time PCR.

	**Forward (5´ - 3´)**	**Reverse (5´ - 3´)**
18S	gtaacccgttgaaccccatt	ccatccaatcggtagtagcg
Mtr1	agcgcgccctagttggtctg	gcagcttgcggttcctgagc
Mtr2	agggctaccgtgcctgtcaa	aggtttgctgctaggcccact

Mtr, melatonin receptor. Expressions were normalized to 18S mRNA expression.
