# Supplementary material for: Oral supplementation of melatonin attenuates the onset of alcohol-related liver disease
Source: J Mol Med (Berl). 2025 Aug 7;103(10):1219–30. doi: 10.1007/s00109-025-02583-4 (PMC12449381; doi:10.1007/s00109-025-02583-4)
Supplement: Supplementary file 4 — (PDF 91.3 KB) [file 109_2025_2583_MOESM4_ESM.pdf]

Oral Supplementation of Melatonin Attenuates the Onset of Alcohol-Related Liver Disease

Journal of Molecular Medicine

Franziska Kromm, Anja Baumann, Victor Sánchez, Annette Brandt, Raphaela Staltner, Ina Bergheim*

*** Corresponding author:** Ina Bergheim, Ph.D.
University of Vienna
Department of Nutritional Sciences
Molecular Nutritional Science
Josef-Holaubek-Platz 2 (UZA II)
A-1090 Vienna
E-Mail: ina.bergheim@univie.ac.at

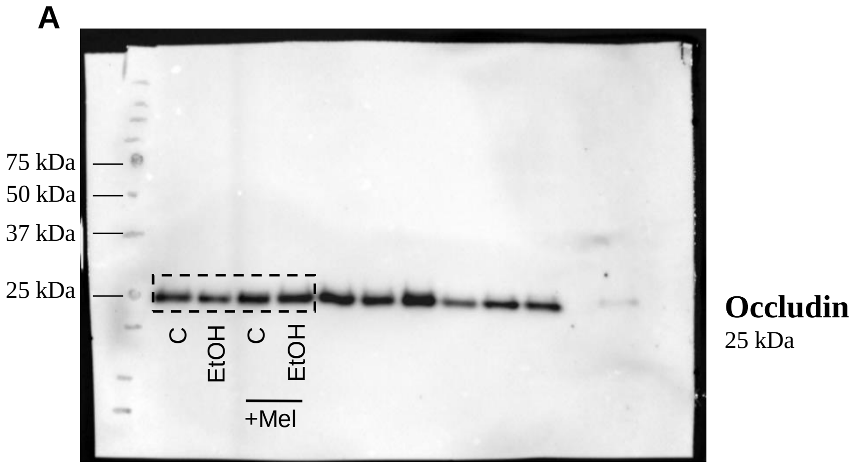

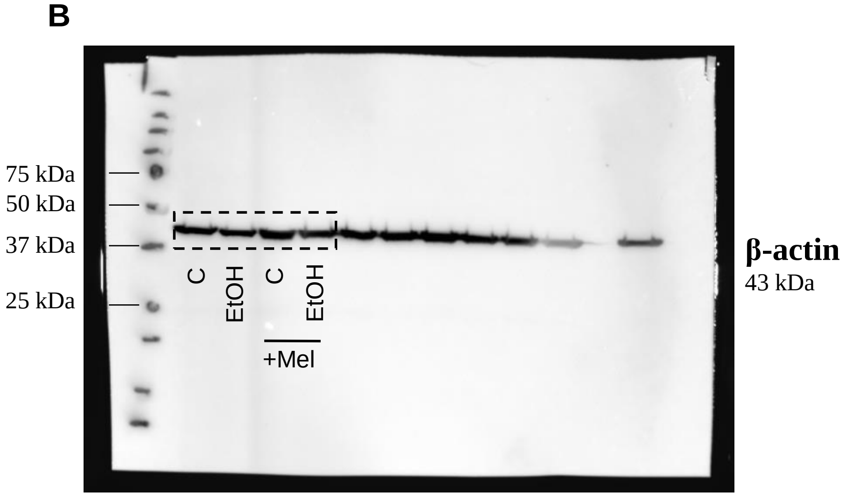

Online Resource 4: Pictures of Western Blot of (A) occludin and (B) β-actin in small intestine of mice fed a alcohol containing or control diet supplemented with melatonin. Bands that are shown in Figure 2E are highlighted with a frame. C: Control diet; EtOH: ethanol-enriched Lieber DeCarli liquid diet; Mel: melatonin.
