# Supplementary material for: Oral supplementation of melatonin attenuates the onset of alcohol-related liver disease
Source: J Mol Med (Berl). 2025 Aug 7;103(10):1219–30. doi: 10.1007/s00109-025-02583-4 (PMC12449381; doi:10.1007/s00109-025-02583-4)
Supplement: Supplementary file 6 — (PDF 125 KB) [file 109_2025_2583_MOESM6_ESM.pdf]

Oral Supplementation of Melatonin Attenuates the Onset of Alcohol-Related Liver Disease

Journal of Molecular Medicine

Franziska Kromm, Anja Baumann, Victor Sánchez, Annette Brandt, Raphaela Staltner, Ina Bergheim*

*** Corresponding author:** Ina Bergheim, Ph.D.
University of Vienna
Department of Nutritional Sciences
Molecular Nutritional Science
Josef-Holaubek-Platz 2 (UZA II)
A-1090 Vienna
E-Mail: ina.bergheim@univie.ac.at

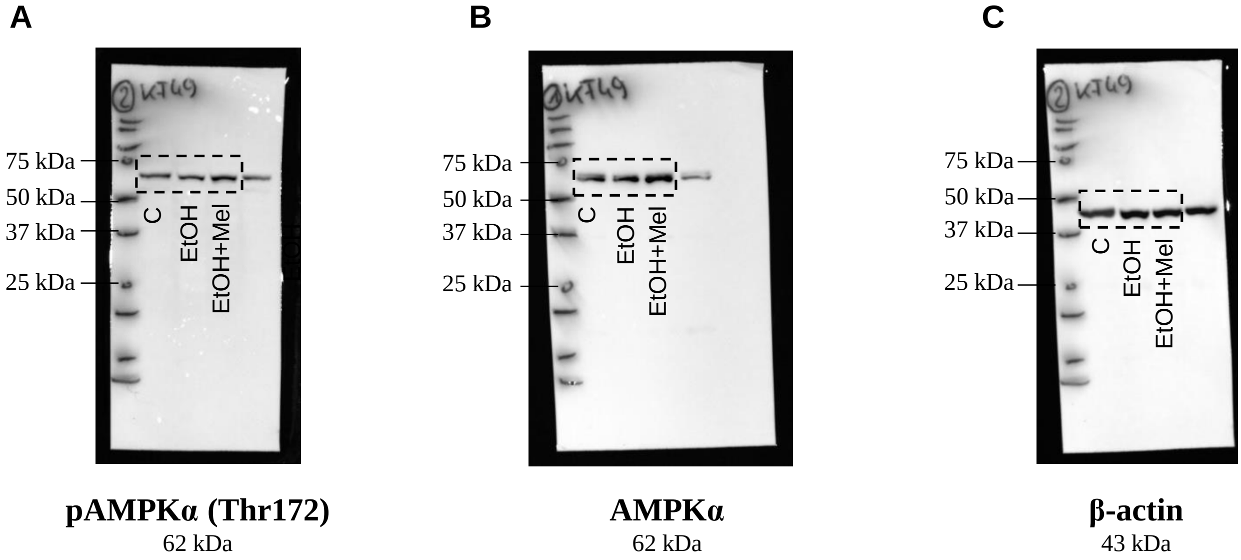

Online Resource 6: Pictures of Western Blot of (A) pAMPKα, (B) AMPKα and (C) β-actin in *ex vivo* small intestinal tissue everted gut sacs challenged with 0.5‰ ethanol ± melatonin. Bands that are shown in Figure 4C are highlighted with a frame. AMPK: 5′-AMP-activated protein kinase; C: control; EtOH: ethanol; Mel: melatonin.
